# Dual-conditioned diffusion model with anatomical guidance for geometric distortion correction in prostate MRI

**DOI:** 10.1186/s41747-026-00735-w

**Published:** 2026-05-13

**Authors:** Inye Na, Qi Miao, Jonghun Kim, Kyunghyun Sung, Hyunjin Park

**Affiliations:** 1 Department of Electrical and Computer Engineering, Sungkyunkwan University, Suwon, Republic of Korea; 2 Department of Radiological Sciences, David Geffen School of Medicine, University of California, Los Angeles, Los Angeles, CA USA

**Keywords:** Artifacts, Artificial intelligence, Deep learning, Diffusion magnetic resonance imaging, Prostate

## Abstract

**Objective:**

Geometric distortion from susceptibility artifacts in diffusion-weighted imaging (DWI) degrades anatomical fidelity and complicates clinical prostate magnetic resonance imaging (MRI) interpretation. We developed and evaluated DeDistortNet, a generative dual-conditioned diffusion model for correcting geometric distortions in prostate DWI without experimentally acquired paired distorted-undistorted data.

**Materials and methods:**

We utilized the public PROSTATEx dataset, divided into training (*n* = 135, 1,893 slices), validation (*n* = 4, 64 slices), and test (*n* = 189, 2,623 slices) cohorts stratified by distortion severity. Model training used only undistorted DWIs, with simulated distortions to generate paired examples. DeDistortNet combines contextual guidance from distorted DWIs with structural guidance from T2-weighted images to synthesize distortion-free DWIs. Performance was evaluated using quantitative analysis on simulated distortions and indirect validation on clinically distorted DWIs through anatomical concordance with T2-derived prostate masks.

**Results:**

The cohort included prostate MRI exams from 328 male subjects. The DWIs from 139 exams were undistorted for training/validation, while 189 were distorted for testing. In simulated data, DeDistortNet restored image quality across distortion severities, improving peak signal-to-noise ratio by 36% and structural similarity by 55% in the peripheral zone (PZ) under extreme distortion. In clinically distorted data, concordance with T2-weighted references (PZ Dice similarity) improved by 37% under severe distortion and by 72% under extreme distortion. Radiologist assessment further supported improved geometric fidelity and prostate boundary delineation after correction.

**Conclusion:**

DeDistortNet, trained on undistorted DWIs with simulated distortions, effectively corrected distortions in prostate DWI and restored anatomical fidelity, particularly in the PZ, without requiring additional acquisitions or specialized imaging protocols.

**Relevance statement:**

By correcting severe distortions without additional imaging sequences, DeDistortNet restores anatomical fidelity in prostate diffusion-weighted imaging, particularly in the peripheral zone, enabling more reliable image interpretation and reducing the need for repeat scans.

**Key Points:**

- DeDistortNet corrects geometric distortion in prostate diffusion MRI.
- Our method integrates anatomical guidance from T2-weighted images for distortion-free reconstruction.
- Our method was trained on simulated distortions without experimentally acquired paired distorted-undistorted data or additional MRI sequences.

**Graphical Abstract:**

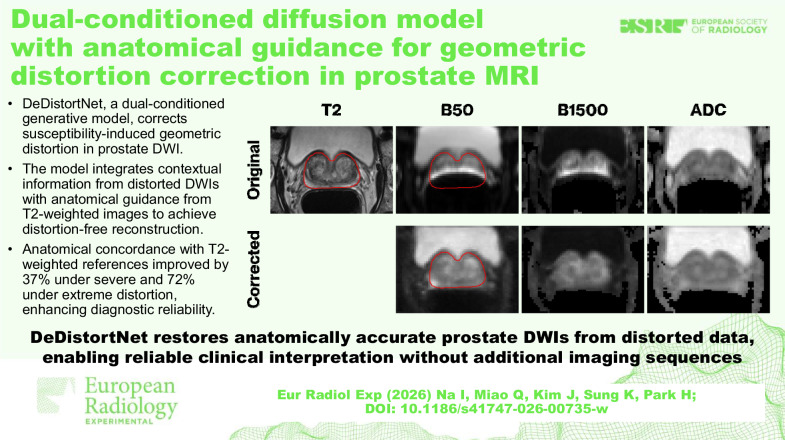

## Background

Prostate diffusion-weighted imaging (DWI) is typically acquired using single-shot echo-planar imaging (EPI), whose geometric distortion remains a persistent challenge [1–3]. Susceptibility-induced distortions cause DWI images to appear stretched, compressed, or displaced relative to anatomic position, resulting in significant misalignment with T2-weighted images. These distortions compromise anatomical fidelity, potentially affecting clinical interpretation and image analysis.

Several methods have been developed to reduce geometric distortion in EPI-based DWI. Field map-based techniques measure B_0_ inhomogeneity using additional gradient-echo sequences to calculate voxel shift maps for precise correction [1, 4]. Another approach acquires image pairs with opposite phase-encoding directions (blip-up/blip-down) to directly estimate deformation fields from reversed distortion patterns [5, 6]. Multi-shot or readout-segmented EPI techniques minimize susceptibility-induced distortion by shortening echo trains during acquisition [7–11]. Distortion can also be reduced by using a reduced field of view along the phase-encoding direction, which is less sensitive to off-resonance effects [12]. However, these conventional methods often require additional imaging sequences or specialized acquisition protocols, making them impractical for routine clinical prostate MRI due to scan time constraints and patient motion.

As an alternative, registration-based methods attempt distortion correction by aligning distorted DWIs to anatomically accurate references (*e.g*., T2-weighted images) using rigid or deformable transformations [13–16]. While these methods do not require additional acquisitions, they often fail under severe distortion conditions. Residual susceptibility artifacts can lead to substantial signal pile-ups or voids that intensity-driven registration methods alone cannot reliably recover [17]. Supplementary Fig. S1 illustrates these limitations, providing an example of severe prostate DWI distortion where traditional nonrigid registration (*e.g*., advanced normalization tools) fails to adequately correct anatomical displacement and signal artifacts.

Despite significant advances in deep learning applications for prostate MRI, methods specifically addressing severe geometric distortions in DWI remain underexplored. Recent studies have primarily focused on improving image resolution or signal-to-noise ratio (SNR). Super-resolution and denoising approaches have enhanced image quality but do not directly address geometric distortions [18, 19]. Techniques such as misalignment augmentation or adversarial noise training have been used to increase robustness against distortions, but do not explicitly correct visible anatomical distortions [20, 21]. Thus, a clear unmet need remains for deep learning methods that can directly correct severe susceptibility-induced geometric distortions in prostate DWI.

To address this challenge, we introduce DeDistortNet, a generative deep learning model named to reflect its function of removing (“De-”) distortion (“Distort”), specifically designed to correct geometric distortions in prostate DWI. Unlike deformation field estimation methods, DeDistortNet directly synthesizes undistorted DWI images guided by corresponding T2-weighted images. The model is trained using distorted images simulated from originally undistorted DWIs, enabling robust training on anatomically accurate images even under severe distortion and significant signal disruption. We evaluate DeDistortNet as a potential solution for correcting geometric distortion in routinely acquired prostate MRI.

## Methods

### Study population and image acquisition

This retrospective study utilized the publicly available, fully anonymized PROSTATEx dataset [22]. Institutional Review Board approval and informed consent were obtained by the dataset collectors. Prostate MRI was performed on two 3-T MRI systems (MAGNETOM Trio and Skyra, Siemens Healthineers) without an endorectal coil. T2-weighted images were acquired in axial orientation using a turbo spin-echo sequence with 0.5-mm in-plane resolution and 3-mm slice thickness. DWIs were obtained in axial orientation using single-shot EPI with a 2 mm in-plane resolution, 3-mm slice thickness, and diffusion-encoding gradients in three orthogonal directions (*b*-values: 50, 400, and 800 s/mm^2^). Although TCIA documentation reports a 3.6 mm slice thickness, Digital Imaging and Communications in Medicine (DICOM) header inspection showed that most T2-weighted and DWI scans were acquired with a 3.0-mm slice thickness (T2-weighted: 95%; DWI: 88%). Prostate masks were derived from exams matched to the Prostate Imaging–Cancer AI dataset [23]. Nineteen unmatched exams were excluded. Clinical data, including patient age, prostate-specific antigen (PSA), PSA density (PSAD), prostate volume, International Society of Urological Pathology‒ISUP grades, and clinically significant prostate cancer, were also retrieved from PICAI.

### Data preparation and distortion labeling

All DWIs were first resampled to the corresponding T2-weighted image space and standardized to a voxel spacing of $0.5\times 0.5\times 3$ mm. To focus on the prostate and adjacent tissues, images were cropped to twice the size of the prostate region.

To systematically characterize distortion severity, each DWI slice was assessed using rule-based criteria, with severity determined by the mismatch (in millimeters) between the T2-derived prostate boundary and its appearance on DWI. Severity was assigned as no distortion, mild (< 2 mm), moderate (2–4 mm), severe (4–6 mm), or extreme (> 6 mm). The initial grading was performed by a graduate student in engineering and was subsequently confirmed by the senior author with 14 years of experience in prostate MRI. Representative examples across distortion severity levels are provided in the “Qualitative evaluation” section.

**Fig. 1:**
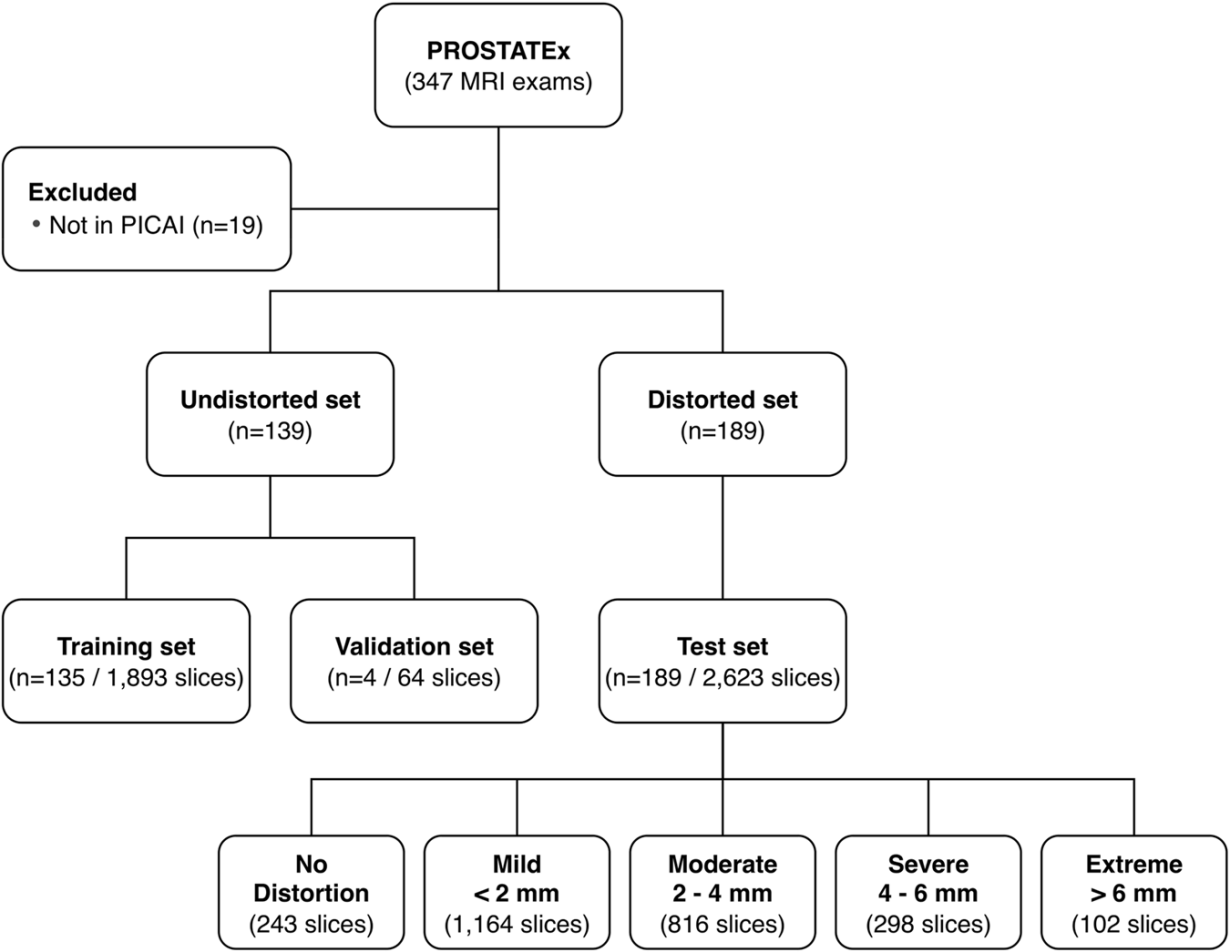
Flowchart of subject inclusion and dataset stratification by distortion severity

Exams without any distorted slices were assigned to the training and validation sets, while exams containing at least one distorted slice were allocated to the test set. This classification yielded training (*n* = 135; 1,893 slices), validation (*n* = 4; 64 slices), and test (*n* = 189; 2,623 slices) cohorts. Test set slices were further stratified by distortion severity: no distortion (243 slices), mild (1,164 slices), moderate (816 slices), severe (298 slices), and extreme (102 slices). As the test set was defined at the subject level, some exams included slices with no visible distortion, leading to “no distortion” slices within the distorted cohort. A flowchart providing an overview of subject inclusion and dataset stratification is presented in Fig. 1.

Finally, min-max normalization parameters were derived from intensity values within the prostate regions of the training set and applied consistently to the validation and test sets.

### Distortion simulation algorithm

In routine clinical prostate MRI, it is not feasible to acquire paired distorted and undistorted DWIs, as this would require additional sequences or specialized acquisition protocols. To overcome this limitation, we established a simulation framework in which realistic distortion patterns were randomly applied to distortion-free training slices, enabling the model to learn correction strategies across a broad range of susceptibility-related appearances.

Susceptibility-induced EPI distortion is fundamentally driven by B0 inhomogeneity and is commonly described as displacement along the phase-encoding direction. However, because B0 inhomogeneity varies spatially across the field of view, the resulting displacement is location-dependent and appears nonuniform, often accompanied by signal pile-ups or voids in regions with strong susceptibility effects [1, 24]. Accordingly, we incorporated two-dimensional elastic deformation within the rectal-adjacent prostate region to approximate spatially varying warping, together with Jacobian-based intensity modulation to mimic signal pile-up/void behavior. In addition, rigid transformations (scaling, rotation, and translation) were used as practical augmentations to improve robustness to residual misalignment and acquisition variability.

**Fig. 2:**
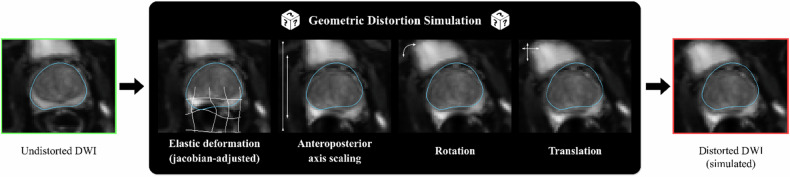
Detailed steps of the geometric distortion simulation applied to originally undistorted diffusion-weighted images, including elastic deformation (Jacobian-adjusted), anteroposterior axis scaling, rotation, and translation

Distortion simulations during model training comprised four sequential, randomly applied transformations: elastic deformation with Jacobian intensity adjustment, anteroposterior axis scaling, rotation, and translation (Fig. 2).

Elastic deformation was selectively applied to the posterior prostate region adjacent to the rectum by generating random displacement fields ${{\rm{u}}}({{\rm{x}}},{{\rm{y}}})$ on a coarse grid (spacing = 32 pixels; 16 mm). Outside these targeted regions, displacement vectors were set to zero. Within the targeted regions, displacement vectors were defined as:

$${{\rm{u}}}\left({{\rm{x}}},{{\rm{y}}}\right)=\left[{{{\rm{u}}}}_{{{\rm{x}}}}\left({{\rm{x}}},{{\rm{y}}}\right),{{{\rm{u}}}}_{{{\rm{y}}}}\left({{\rm{x}}},{{\rm{y}}}\right)\right],\,{{{\rm{u}}}}_{{{\rm{x}}}}\left({{\rm{x}}},{{\rm{y}}}\right),\,{{{\rm{u}}}}_{{{\rm{y}}}}\left({{\rm{x}}},{{\rm{y}}}\right){{\mathscr{ \sim }}}{{\mathscr{N}}}\left(0,{{{\rm{\sigma }}}}^{2}\right),$$

where ${{\rm{\sigma }}}$ was empirically set to 10 pixels, generating randomized displacement fields that produced diverse distortions. These coarse displacement fields were smoothly interpolated onto the original image resolution, inducing clinically realistic local expansions or contractions [25, 26].

The Jacobian determinant ${{\rm{J}}}({{\rm{x}}},{{\rm{y}}})$, representing local volumetric changes induced by deformation, was computed as:

$${{\rm{J}}}\left({{\rm{x}}},{{\rm{y}}}\right)=\left(1+\frac{\partial {{{\rm{u}}}}_{{{\rm{x}}}}\left({{\rm{x}}},{{\rm{y}}}\right)}{\partial {{\rm{x}}}}\right)\left(1+\frac{\partial {{{\rm{u}}}}_{{{\rm{y}}}}\left({{\rm{x}}},{{\rm{y}}}\right)}{\partial {{\rm{y}}}}\right)-\frac{\partial {{{\rm{u}}}}_{{{\rm{x}}}}\left({{\rm{x}}},{{\rm{y}}}\right)}{\partial {{\rm{y}}}}\frac{\partial {{{\rm{u}}}}_{{{\rm{y}}}}\left({{\rm{x}}},{{\rm{y}}}\right)}{\partial {{\rm{x}}}}.$$

Intensity adjustment was performed to simulate realistic voxel intensity variations (signal pile-ups or voids) resulting from geometric distortion. The distorted image ${{{\rm{I}}}}_{{{\rm{distorted}}}}({{\rm{x}}},{{\rm{y}}})$ was computed as:

$${{{\rm{I}}}}_{{{\rm{distorted}}}}\left({{\rm{x}}},{{\rm{y}}}\right)={{{\rm{I}}}}_{{{\rm{elastic}}}\; {{\rm{deformed}}}}\left({{\rm{x}}},{{\rm{y}}}\right)\cdot \left|{{\rm{J}}}\left({{\rm{x}}},{{\rm{y}}}\right)\right|.$$

Finally, random scaling along the anteroposterior axis (scale factor: $0.9-1.1$), random rotations (± $15^\circ$), and random translations (± $2$ mm in-plane) were applied to enhance distortion realism. During training, these transformations were applied as continuously varying distortions rather than predefined severity levels, allowing the model to learn from a broad range of artifact patterns.

### DeDistortNet model architecture and training

**Fig. 3:**
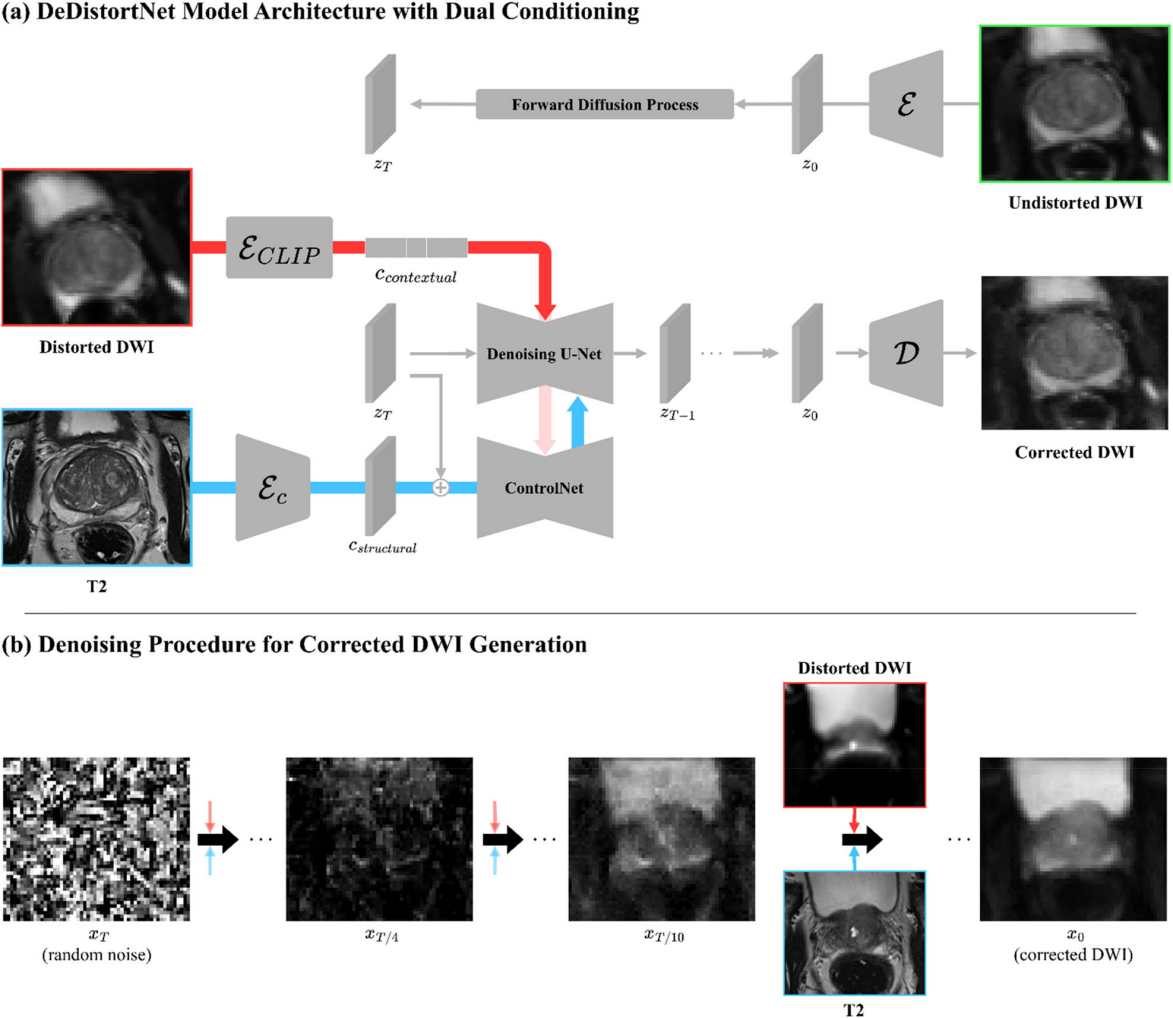
Model architecture of DeDistortNet and denoising workflow. **a** Model architecture showing the dual-conditioning strategy, incorporating structural guidance from T2-weighted images and contextual guidance from distorted diffusion-weighted images. **b** Step-by-step denoising procedure demonstrating the generation of corrected diffusion-weighted images from initial random noise conditioned on distorted diffusion-weighted images and corresponding T2-weighted images

DeDistortNet is a generative latent diffusion model [27] specifically designed to correct geometric distortion in prostate DWIs. This generation-based approach synthesizes distortion-free DWIs by combining two complementary sources of information: contextual guidance from distorted DWIs and structural (*i.e*., anatomical) guidance from corresponding T2-weighted images (Fig. 3).

We selected Stable Diffusion, a generative model originally developed for text-to-image synthesis, as our backbone framework due to its established pretrained variational autoencoder [27]. This variational autoencoder comprises an encoder (${{\varepsilon}}$) and a decoder (${{\mathcal{D}}}$), efficiently compressing high-dimensional images into low-dimensional latent representations. This latent-based approach enables stable training, reduces computational complexity, and facilitates robust synthesis of high-quality images, making it particularly suitable for medical imaging applications.

In standard Stable Diffusion, text prompts encoded by a pretrained contrastive language-image pretraining (CLIP) text encoder guide image synthesis through cross-attention mechanisms. Since our task involves correcting image distortion rather than generating images from text, we replaced the CLIP text encoder with a pretrained CLIP image encoder (${{{\varepsilon}}}_{{{\rm{CLIP}}}}$) [28]. The image encoder was initialized from pretrained weights originally trained on natural images and further adapted during DeDistortNet training using our training data with simulated distortions. This modification provides a contextual embedding (${{{\rm{c}}}}_{{{\rm{contextual}}}}$) from distorted DWIs, capturing intrinsic signal characteristics such as diffusion-weighted signal intensities across various *b*-values. To ensure the contextual embedding primarily encodes authentic DWI signal patterns rather than distortion-induced artifacts, randomized distortion simulations—including elastic deformation, scaling, rotation, and translation—were applied during training (Supplementary Fig. S2b).

Synthesizing distortion-free DWIs requires anatomical consistency. To explicitly enforce accurate anatomical structure, we incorporated ControlNet, a recent technique designed to provide structural guidance to diffusion models [29]. Using a dedicated ControlNet encoder (${{{\varepsilon}}}_{{{\rm{c}}}}$), we obtained a structural embedding (${{{\rm{c}}}}_{{{\rm{structural}}}}$) from corresponding T2-weighted images. Unlike contextual embedding, which guides intensity patterns *via* cross-attention layers, structural embedding directly modulates intermediate feature maps within the denoising U-Net. This direct structural guidance enables our method to preserve accurate prostate anatomy during image synthesis.

Diffusion models generally consist of two main processes: a forward diffusion process, in which Gaussian noise is incrementally added to progressively corrupt images; and a reverse diffusion (denoising) process, in which this noise is iteratively removed to reconstruct the original images. DeDistortNet follows this two-step procedure (Fig. 3a). First, undistorted DWIs are encoded into latent representations (${{\rm{z}}}_{0}={{\varepsilon}}({{\rm{x}}}_{0})$), which are then progressively corrupted into increasingly noisy latent vectors (${{{\rm{z}}}}_{1},\,\ldots ,\,{{{\rm{z}}}}_{{{\rm{T}}}}$) during forward diffusion. Subsequently, in the reverse diffusion process, the model iteratively removes noise at each timestep ${{\rm{t}}}$, guided simultaneously by contextual and structural embeddings. Model parameters are optimized by minimizing the difference between the predicted noise (${\hat{{{\rm{\epsilon }}}}}_{{{\rm{\theta }}}}$) and the actual noise (${{\rm{\epsilon }}}$) added during the forward diffusion process:

$${{\mathcal{L}}}\left({{\rm{\theta }}}\right)={{\mathbb{E}}}_{{{{\rm{z}}}}_{0},{{\rm{\epsilon }}},{{\rm{t}}}}\left[{{||}{{\rm{\epsilon }}}-{\hat{{{\rm{\epsilon }}}}}_{{{\rm{\theta }}}}\left({{{\rm{z}}}}_{{{\rm{t}}}},{{\rm{t}}},{{{\rm{c}}}}_{{{\rm{contextual}}}},{{{\rm{c}}}}_{{{\rm{structural}}}}\right){||}}_{2}^{2}\right].$$

After denoising, the resulting latent representation (${{{\rm{z}}}}_{0}$) is decoded back into the original image space (${{\rm{x}}}_{0}={{\mathscr{D}}}({{\rm{z}}}_{0})$), yielding geometrically corrected DWIs that accurately reflect realistic diffusion-weighted intensities while maintaining precise anatomical structures.

Both distorted and corrected DWIs consisted of three diffusion-weighted channels (*b*-values: 50, 400, and 800 s/mm^2^), and T2-weighted inputs were single-channel. Images were zero-padded to achieve a consistent dimension of $512\times 512$ pixels. Training was performed using the AdamW optimizer with a batch size of 4 and an initial learning rate of $1\times {10}^{-5}$.

### Comparison models

Comparison models included AdaIN [30], AdaConv [31], pix2pix [32], CycleGAN [33], ControlNet [29], and Stable Diffusion [27]. AdaIN and AdaConv are GAN-based models capable of using both DWI and T2-weighted images as inputs, similar to DeDistortNet. Specifically, distorted DWIs served as style images, while corresponding T2-weighted images were used as content images during training, consistent with the original implementations of these methods. Pix2pix and CycleGAN, translation-based GAN models, utilized only T2-weighted images as input. ControlNet retained the original CLIP text encoder, using only T2-weighted images with the text prompt: “Rectal artifact-free DWI images for *b*-values 50, 400, and 800 s/mm^2^ with anatomical guidance from T2-weighted images.” Stable Diffusion used only distorted DWI inputs with a CLIP image encoder, without ControlNet.

### Evaluation metrics

Quantitative evaluation metrics included Peak Signal-to-Noise Ratio (PSNR), structural component of the Structural Similarity Index Measure (S-SSIM), and Dice coefficient. PSNR evaluates similarity between reference and reconstructed images by measuring pixel intensity differences [34]; higher PSNR values indicate superior image quality. S-SSIM specifically quantifies local structural consistency while being less influenced by global luminance or contrast variations [34], thereby reflecting preservation of clinically relevant anatomical details. The dice coefficient measures spatial overlap between segmented regions, with values ranging from 0 (no overlap) to 1 (perfect overlap), quantifying segmentation accuracy [35].

### Evaluation on actual distorted data

As the dataset does not include paired undistorted DWIs, direct evaluation of distortion correction on clinically distorted DWIs is not feasible. Instead, an indirect strategy is employed by leveraging T2-weighted images as anatomically reliable references. The DWI masks are obtained using nnUNet [36], a deep learning segmentation model trained on the same distortion-free DWIs used in the training set, with corresponding T2-weighted masks as ground truth. The trained model is applied to DWIs before and after correction to obtain DWI masks, and Dice coefficients are computed against the corresponding T2-weighted masks. Dice evaluation is performed for both the prostate region and the peripheral zone (PZ), and results are summarized by distortion severity.

Mask generation is highly dependent on realistic intensity distributions, and inadequate correction would result in degraded mask quality. Improvements in Dice coefficients after correction therefore serve as an indirect validation of both intensity fidelity and anatomical accuracy.

### Reader study

A radiologist reader study was performed to evaluate image quality before and after distortion correction. A volumetric evaluation set was constructed by selecting 20 exams across distortion severity (five exams each for mild, moderate, severe, and extreme), where volumetric severity was defined as the maximum slice-level distortion severity within each exam. One board-certified abdominal radiologist reviewed DWI datasets acquired before and after correction, with axial T2-weighted images provided as anatomical reference. For each dataset, the *b*50 image, the estimated *b*1500 image, and the corresponding apparent diffusion coefficient (ADC) map were provided, consistent with the estimation procedure described in the subsection “DeDistortNet model architecture and training.” Datasets were anonymized to maintain blinding with respect to correction status. The reader scored geometric distortion and anatomic delineation on five-point ordinal scales (lower scores indicate better quality) and assessed the need for DWI reacquisition using predefined criteria (Supplementary Table S1) informed by a prior prostate DWI image-quality assessment study [37].

## Results

### Data characteristics

Data characteristics between the undistorted (training and validation sets) and distorted (test set) groups showed no significant differences (Table 1).

**Table 1:** Clinical characteristics of the study population, including patient age, prostate-specific antigen, prostate-specific antigen density, prostate volume, ISUP grades, and clinically significant prostate cancer

	All (*n* = 328)	Undistorted (*n* = 139, 42.4%)	Distorted (*n* = 189, 57.6%)	*p*-value
Age (years)	63.31 ± 7.16	63.33 ± 7.63	63.29 ± 6.81	0.473*
PSA (ng/mL)^†^	14.03 ± 9.88	13.15 ± 9.46	14.68 ± 10.16	0.149*
PSAD (ng/mL^2^)^‡^	0.24 ± 0.19	0.22 ± 0.18	0.26 ± 0.19	0.140*
Prostate volume (mL)^§^	67.64 ± 35.89	69.93 ± 41.06	65.95 ± 31.55	0.835*
ISUP, *N* (%)				0.290^#^
0	177 (54.0)	79 (56.8)	98 (51.9)	
1	40 (12.2)	19 (13.7)	21 (11.1)	
2	57 (17.4)	24 (17.3)	33 (17.5)	
3	29 (8.8)	12 (8.6)	17 (9.0)	
4	13 (4.0)	2 (1.4)	11 (5.8)	
5	12 (3.7)	3 (2.2)	9 (4.8)	
csPCa, *n* (%)	111 (33.8)	41 (29.5)	70 (37.0)	0.191^#^

Statistical comparisons were performed between the undistorted (training and validation) and distorted (test) groups, with no significant differences observed. Data are presented as means ± standard deviations or absolute frequencies with percentages in parentheses

*csPCa* Clinically significant prostate cancer, *ISUP* International Society of Urological Pathology, *PSA* Prostate-specific antigen, *PSAD* Prostate-specific antigen density

^*^ Mann–Whitney *U* test

^#^ χ^2^ test

^†^ PSA missing: 1 in the undistorted group and 3 in the distorted group

^‡^ PSAD missing: 8 in the undistorted group and 17 in the distorted group

^§^ Prostate volume missing: 1 in the undistorted group and 2 in the distorted group

### Performance on the simulated distorted data

**Fig. 4:**
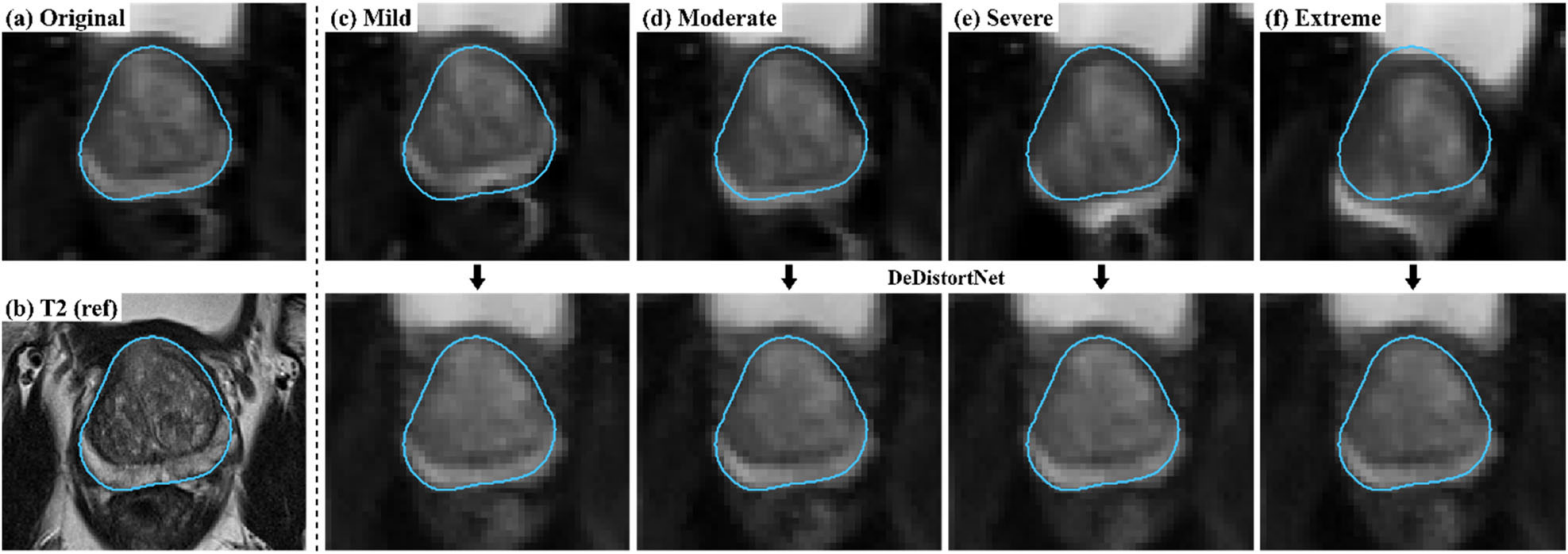
Representative examples of simulated distortion severity and DeDistortNet correction. **a** A distortion-free original test slice. **b** The corresponding T2-weighted image, provided as the anatomical reference. **c**–**f** The same slice with simulated distortions of increasing severity: (**c**) mild, (**d**) moderate, (**e**) severe, and (**f**) extreme (top row). The bottom row in (**c**–**f**) shows the corresponding DeDistortNet-corrected outputs. The cyan contour indicates the prostate mask derived from the T2-weighted image.

As paired distorted and undistorted images are not obtainable in routine clinical prostate MRI, simulated distortions were used to generate artificial paired datasets for evaluation. The same simulation framework applied during model training was employed to ensure methodological consistency. For evaluation, 243 originally distortion-free test slices were each transformed into four versions representing mild, moderate, severe, and extreme distortions. Parameter ranges were systematically varied for each severity level: elastic deformation (${{\rm{\sigma }}}=3,\,5,\,7,\,10$), anteroposterior axis scaling (0:95–1:05; 0:95–0:99 or 1:01–1:05; 0:90–0:95 or 1:05–1:10; 0:85–0:95 or 1:05–1:15), rotation ($0--3^{\circ}$, $1--3^{\circ}$; $3--5^{\circ}$; $5--15^{\circ}$), and in-plane translation ($\sim 0.5$; 0.5–1; 1–1.5; 1.5–2 mm). Figure 4 shows a representative distortion-free test slice and the corresponding simulated distortions across severity levels, with the T2-weighted image provided as the anatomical reference.

Quantitative evaluation was performed using PSNR and S-SSIM. Metrics were calculated within the prostate and PZ using T2-derived masks, with a subset of 203 slices specifically analyzed for the PZ. Baseline reconstruction performance was also assessed by providing original DWIs as model inputs, confirming the ability to faithfully reproduce distortion-free images in the absence of artifacts.

Table 2 summarizes the performance comparison between DeDistortNet and other generative models (AdaIN, AdaConv, and Stable Diffusion). Models relying solely on T2-weighted images (pix2pix, CycleGAN, and ControlNet) were excluded from this evaluation. DeDistortNet achieved baseline PSNR and S-SSIM values of 19.41 and 0.74 for the prostate, and 17.99 and 0.73 for the PZ. Under simulated distortions, the model consistently outperformed comparison methods. In the PZ, severe distortion improved from 14.06 to 17.97 in PSNR (+28%) and from 0.45 to 0.73 in S-SSIM (+62%). Extreme distortion improved from 13.23 to 17.99 in PSNR (+36%) and from 0.47 to 0.73 in S-SSIM (+55%). These results demonstrate that DeDistortNet effectively corrected pronounced distortions, restoring image quality and anatomical consistency. Mann–Whitney U tests further confirmed that DeDistortNet significantly outperformed AdaIN, AdaConv, and Stable Diffusion across all severity levels (*p* < 0.001), underscoring its robustness and reliability.

**Table 2:** Performance comparison between DeDistortNet and baseline generative models (AdaIN, AdaConv, Stable Diffusion) on simulated distortion severities within prostate and peripheral zone regions

		Baseline (reconstruction)		Mild distortion		Moderate distortion		Severe distortion		Extreme distortion	
		PSNR	S-SSIM	PSNR	S-SSIM	PSNR	S-SSIM	PSNR	S-SSIM	PSNR	S-SSIM
Simulated baseline	Prostate (*n* = 243)	.	.	23.11 ± 5.79	0.70 ± 0.19	21.19 ± 4.85	0.65 ± 0.18	16.21 ± 3.48	0.51 ± 0.15	14.89 ± 3.95	0.50 ± 0.17
	PZ (*n* = 203)	.	.	20.13 ± 5.51	0.65 ± 0.22	18.20 ± 4.64	0.58 ± 0.19	14.06 ± 3.96	0.45 ± 0.17	13.23 ± 4.22	0.47 ± 0.17
AdaIN	Prostate (*n* = 243)	13.90 ± 3.27	0.58 ± 0.13	13.83 ± 3.28	0.58 ± 0.12	13.85 ± 3.31	0.58 ± 0.13	13.83 ± 3.29	0.58 ± 0.13	13.83 ± 3.30	0.58 ± 0.13
	PZ (*n* = 203)	14.22 ± 3.61	0.53 ± 0.17	14.14 ± 3.60	0.53 ± 0.17	14.16 ± 3.59	0.53 ± 0.17	14.10 ± 3.60	0.52 ± 0.17	14.09 ± 3.52	0.52 ± 0.17
AdaConv	Prostate (*n* = 243)	12.46 ± 2.79	0.59 ± 0.14	12.44 ± 2.80	0.59 ± 0.14	12.43 ± 2.82	0.59 ± 0.14	12.44 ± 2.82	0.59 ± 0.13	12.50 ± 2.83	0.59 ± 0.14
	PZ (*n* = 203)	13.15 ± 3.27	0.53 ± 0.18	13.13 ± 3.31	0.53 ± 0.18	13.13 ± 3.29	0.53 ± 0.18	13.17 ± 3.31	0.53 ± 0.18	13.26 ± 3.36	0.53 ± 0.18
Stable Diffusion	Prostate (*n* = 243)	17.08 ± 4.24	0.67 ± 0.14	17.10 ± 4.30	0.67 ± 0.14	17.12 ± 4.29	0.67 ± 0.14	17.07 ± 4.32	0.67 ± 0.13	16.93 ± 4.14	0.67 ± 0.13
	PZ (*n* = 203)	16.26 ± 4.18	0.65 ± 0.14	16.33 ± 4.23	0.64 ± 0.14	16.38 ± 4.29	0.64 ± 0.14	16.3 ± 4.33	0.64 ± 0.14	16.19 ± 4.49	0.64 ± 0.14
DeDistortNet	Prostate (*n* = 243)	19.41 ± 4.72	0.74 ± 0.11	19.36 ± 4.63	0.74 ± 0.11	19.41 ± 4.62	0.74 ± 0.11	19.36 ± 4.76	0.74 ± 0.11	19.24 ± 4.72	0.74 ± 0.11
	PZ (*n* = 203)	17.99 ± 4.58	0.73 ± 0.12	18.04 ± 4.55	0.72 ± 0.13	18.07 ± 4.59	0.73 ± 0.12	17.97 ± 4.62	0.73 ± 0.12	17.99 ± 4.88	0.73 ± 0.12

Data are mean ± standard deviations. Higher peak signal-to-noise ratio and structural component of the structural similarity index measure values indicate superior correction quality. Each distortion severity category (mild, moderate, severe, extreme) included 243 simulated test images. Mann–Whitney *U* tests showed significantly superior performance of DeDistortNet compared to each baseline model across all severities (*p* < 0.001)

*PSNR* Peak signal-to-noise ratio, *PZ* Peripheral zone, *S-SSIM* Structural component of the structural similarity index measure

### Performance on actual distorted data

As described in “Evaluation on actual distorted data” section, an indirect strategy leveraging T2-weighted images as anatomically reliable references was used to evaluate performance on clinically distorted data. DeDistortNet substantially improved Dice coefficients in the prostate region, from 0.83 to 0.92 (+11%) under severe distortion and from 0.73 to 0.93 (+27%) under extreme distortion (Supplementary Table S2). Gains were even more pronounced in the PZ, increasing from 0.57 to 0.78 (+37%) under severe distortion and from 0.46 to 0.79 (+72%) under extreme distortion (Table 3).

**Table 3:** Dice similarity coefficients comparing peripheral zone masks obtained from T2-weighted images and distorted/corrected diffusion-weighted images across varying distortion severities

	No distortion (*n* = 203)	Mild distortion (*n* = 976)	Moderate distortion (*n* = 710)	Severe distortion (*n* = 259)	Extreme distortion (*n* = 86)	Average (*n* = 2,234)
Baseline	0.78 ± *0.22*	0.75 ± 0.22*	0.64 ± 0.24*	0.57 ± 0.25*	0.46 ± 0.27*	0.69 ± 0.25*
AdaIN	0.16 ± 0.36*	0.16 ± 0.36*	0.13 ± 0.33*	0.13 ± 0.34*	0.15 ± 0.35*	0.15 ± 0.35*
AdaConv	0.16 ± 0.36*	0.16 ± 0.37*	0.13 ± 0.33*	0.13 ± 0.33*	0.16 ± 0.36*	0.15 ± 0.35*
pix2pix	0.63 ± 0.31*	0.66 ± 0.29*	0.66 ± 0.29*	0.65 ± 0.31*	0.71 ± 0.26 (*p* = 0.001)	0.66 ± 0.30*
CycleGAN	0.41 ± 0.30*	0.44 ± 0.30*	0.45 ± 0.30*	0.44 ± 0.31*	0.52 ± 0.29*	0.44 ± 0.30*
ControlNet	0.69 ± 0.28*	0.71 ± 0.28*	0.71 ± 0.26*	0.72 ± 0.26 (*p* = 0.001)	0.73 ± 0.27 (*p* = 0.078)	0.71 ± 0.27*
StableDiffusion	0.66 ± 0.27*	0.64 ± 0.26*	0.65 ± 0.25*	0.66 ± 0.25*	0.68 ± 0.24*	0.65 ± 0.26*
DeDistortNet	0.77 ± 0.24 (*p* = 0.873)	0.78 ± 0.24 (*p* = 0.419)	0.78 ± 0.24 (*p* = 0.351)	0.78 ± 0.24 (*p* = 0.166)	0.79 ± 0.22 (*p* = 0.983)	0.78 ± 0.24 (*p* = 0.358)

Asterisks in each severity column indicate statistically significant differences (*p* < 0.001) *versus* the baseline no-distortion group based on Mann–Whitney *U* tests. Asterisks in the “Average” column indicate significant distributional differences across distortion severity groups based on Kruskal–Wallis tests

Statistical analysis showed no significant differences between DeDistortNet-corrected DWIs across all severity groups and the no-distortion group in prostate and PZ Dice distributions using Mann–Whitney U tests (*p* > 0.05). In addition, Kruskal–Wallis tests revealed that baseline results before correction and all other comparison models exhibited significant distributional differences across distortion severity groups (*p* < 0.001), whereas DeDistortNet showed no such differences after correction (prostate: *p* = 0.015; PZ: *p* = 0.358).

### Qualitative evaluation of corrected DWIs

**Fig. 5:**
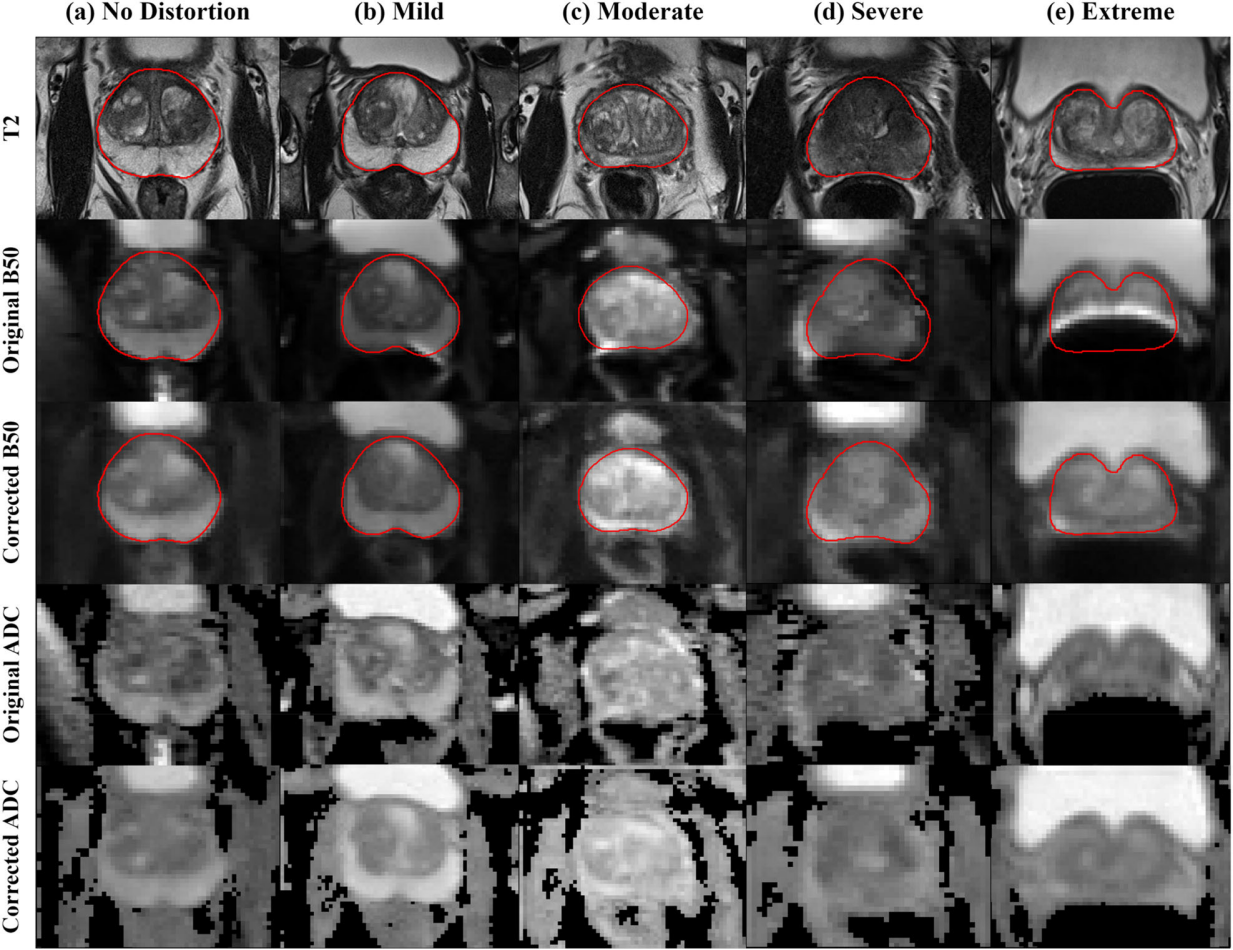
Representative exams across distortion severity levels. Each column shows a different subject from the test set, including (**a**) no distortion, (**b**) mild, (**c**) moderate, (**d**) severe, and (**e**) extreme distortion severity levels. Rows show the T2-weighted reference, distorted B50 diffusion-weighted images, DeDistortNet-corrected B50 diffusion-weighted images, and the corresponding apparent diffusion coefficient map. The red contour indicates the prostate mask derived from the T2-weighted image

Figure 5 provides representative test set exams from different subjects across distortion severity. Each exam includes T2-weighted, distorted B50, corrected B50, and the corresponding apparent diffusion coefficient ADC map.

High *b*-value images (B1500) and ADC maps were estimated from model-generated DWIs at *b*-values of 50, 400, and 800 s/mm^2^ using a mono-exponential diffusion model. ADC and the initial signal intensity without diffusion weighting (S_0_) were estimated by fitting signal intensities at each pixel in logarithmic space using linear least-squares regression. The estimated B1500 image was calculated using ADC and S_0_ as follows:

$${{\rm{S}}}\left({{\rm{b}}}\right)={{{\rm{S}}}}_{0}\exp \left(-{{\rm{b}}}\cdot {{\rm{ADC}}}\right).$$

Supplementary Figures S3 and S4 show qualitative comparisons with other generative models for an undistorted exam and an exam with extreme distortion, respectively, including B50, estimated B1500, and estimated ADC maps.

### Performance of the reader study

The reader study revealed that the geometric distortion score decreased from 3.15 ± 1.04 to 1.45 ± 0.60 after correction across 20 exams. The anatomic delineation score also decreased from 2.95 ± 0.89 to 1.65 ± 0.49, indicating improved prostate boundary delineation. Improvements were consistent across severity groups. Severe and extreme distortion groups showed geometric distortion score reductions from 3.6 to 1.8 and anatomic delineation score reductions from 3.2–3.4 to 1.8. Exam-level comparisons (before *versus* after) for all three endpoints, including reacquisition decisions, are shown in Supplementary Fig. S5. Overall, these results suggest that the proposed method reduces susceptibility-related distortion and improves prostate boundary delineation on DWI, with the potential to reduce the need for repeat acquisitions.

## Discussion

This study evaluated DeDistortNet, a generative dual-conditioned diffusion model, for geometric distortion correction in prostate DWI acquired with single-shot EPI. The model improved image quality across distortion severities and demonstrated particularly pronounced benefits in the peripheral zone (PZ), which is highly vulnerable to rectal susceptibility artifacts. In this region, Dice concordance with T2-weighted anatomical references improved by 37% under severe distortion and by 72% under extreme distortion, indicating substantial recovery of anatomical fidelity. These quantitative findings were complemented by radiologist assessment, which showed reduced geometric distortion and improved prostate boundary delineation after correction across severity levels. Overall, these results support the potential of DeDistortNet to enhance prostate DWI interpretability without additional acquisitions. This is particularly relevant as recent efforts to improve prostate DWI through ultrahigh-gradient acquisitions have not shown diagnostic benefit over standard b-values [38]. From a deployment perspective, although model training was performed on a single NVIDIA A100 GPU (80 GB), inference can be executed with approximately 6 GB of GPU memory, which is commonly available on clinical workstation GPUs. The average inference time was approximately 6 s per slice.

Compared with established generative approaches, DeDistortNet achieved superior performance across distortion severities. Methods relying exclusively on T2-weighted anatomical guidance, such as pix2pix and CycleGAN, were unable to restore diffusion-specific contrast essential for clinical interpretation. Stable Diffusion, trained without anatomical conditioning, generated images with limited structural fidelity. AdaIN and AdaConv, while incorporating both DWI and T2 information, were constrained by dependence on global statistical features. By contrast, DeDistortNet’s dual-conditioning design, integrating distorted DWI signal context with T2-derived structural guidance, enabled anatomically faithful and diffusion-consistent correction even in the presence of severe signal pile-ups and voids.

Several limitations should be acknowledged. First, generalizability may be affected by domain shift because training relied on simulated distortions, and variations across scanners and acquisition protocols were not systematically evaluated. Multicenter validation will be important to establish robustness under diverse acquisition settings. Second, as DeDistortNet is a generative model, there is a potential risk of introducing hallucinated or anatomically implausible structures. Although this was not directly quantified in the current study, the segmentation-based concordance analysis may provide a practical monitoring signal because unrealistic outputs would be expected to reduce agreement with T2-derived prostate masks. Third, distortion severity labeling was based on a rule-based boundary mismatch criterion and was performed by a non-radiologist primary rater with senior author confirmation. Inter-observer variability was not assessed, and future work with multi-reader radiologist grading and reliability analysis will be useful to further validate the robustness of the severity stratification. Finally, distortion severity labeling and model training were performed in a slice-wise (two-dimensional) manner. While this strategy allowed fine-grained supervision for local artifacts, volumetric (three-dimensional) extensions may further enhance the ability to address complex deformations spanning contiguous slices. Separately, as DeDistortNet uses T2-weighted images for structural guidance, ongoing improvements in T2-weighted acquisition quality [39] could further enhance correction performance.

In summary, DeDistortNet integrates contextual and anatomical guidance within a generative diffusion framework to correct geometric distortions in prostate DWI. The marked recovery of structural concordance with T2-weighted references in the PZ underscores its clinical relevance, highlighting its potential to improve diagnostic confidence in prostate MRI. These findings suggest that DeDistortNet could be incorporated into routine workflows to enhance image quality and interpretability without additional acquisitions or protocol modifications.

## Supplementary information

**Additional File 1: Supplementary Fig. S1** Comparison of distortion correction methods. The registration-based approach (*e.g*., advanced normalization tools) failed to correct severe distortion, whereas the proposed generative approach (DeDistortNet) successfully mitigated these artifacts. **Supplementary Fig. S2** Overview of DeDistortNet workflow. (a) Severity labeling for diffusion-weighted images distortion. (b) Training procedure using simulated distorted diffusion-weighted images. (c) Geometric distortion correction using trained DeDistortNet. Supplementary Table S1 Criteria for reader study. **Supplementary Table S2** Dice similarity coefficients comparing prostate masks obtained from T2-weighted images and distorted/corrected diffusion-weighted images across varying distortion severities. **Supplementary Fig. S3** Qualitative comparison of diffusion-weighted images generated by DeDistortNet and comparative generative models in a no-distortion case. Displayed images include the T2-weighted reference, B50, estimated B1500, and estimated ADC maps. The red contour indicates the prostate mask derived from the T2-weighted image. **Supplementary Fig. S4** Qualitative comparison of diffusion-weighted images generated by DeDistortNet and comparative generative models in an extreme distortion case. Displayed images include the T2-weighted reference, B50, estimated B1500, and estimated ADC maps. The red contour indicates the prostate mask derived from the T2-weighted image. **Supplementary Fig. S5** Reader study results across distortion severity. Before *vs*. after comparisons are shown for (a) geometric distortion, (b) anatomic delineation, and (c) need for reacquisition across mild, moderate, severe, and extreme distortion severity. In (a) and (b), lower scores indicate better image quality. In (c), “No*” denotes baseline-level artifact without diagnostic impact (Supplementary Table S2). Circle size is proportional to the number of cases with the same before–after rating combination. **Supplementary Table S1** Criteria for reader study. **Supplementary Table S2** Dice similarity coefficients comparing prostate masks obtained from T2-weighted images and distorted/corrected diffusion-weighted images across varying distortion severities.

## Data Availability

The dataset analyzed during this study is publicly available in The Cancer Imaging Archive (TCIA) at https://www.cancerimagingarchive.net/collection/prostatex/.

## Abbreviations

ADC: Apparent diffusion coefficient
CLIP: Contrastive language-image pretraining
DWI: Diffusion-weighted imaging
EPI: Echo-planar imaging
PSA: Prostate-specific antigen
PSAD: Prostate-specific antigen density
PSNR: Peak signal-to-noise ratio
PZ: Peripheral zone
S-SSIM: Structural component of the structural similarity index measure
TCIA: The Cancer Imaging Archive

## Code availability

The derived slice-level distortion severity labels and model implementation codes developed for this study are publicly available in a dedicated GitHub repository at: https://github.com/nainye/DeDistortNet.
