# Supplementary material for: Dual-conditioned diffusion model with anatomical guidance for geometric distortion correction in prostate MRI
Source: Eur Radiol Exp. 2026 May 13;10:66. doi: 10.1186/s41747-026-00735-w (PMC13172242; doi:10.1186/s41747-026-00735-w)
Supplement: Supplementary file 1 — Additional File 1: Supplementary Fig. S1 Comparison of distortion correction methods. The registration-based approach (e.g., advanced normalization tools) failed to correct severe distortion, whereas the proposed generative approach (DeDistortNet) successfully mitigated these artifacts. Supplementary Fig. S2 Overview of DeDistortNet workflow. (a) Severity labeling for diffusion-weighted images distortion. (b) Training procedure using simulated distorted diffusion-weighted images. (c) Geometric distortion correction using trained DeDistortNet. Supplementary Table S1 Criteria for reader study. Supplementary Table S2 Dice similarity coefficients comparing prostate masks obtained from T2-weighted images and distorted/corrected diffusion-weighted images across varying distortion severities. Supplementary Fig. S3 Qualitative comparison of diffusion-weighted images generated by DeDistortNet and comparative generative models in a no-distortion case. Displayed images include the T2-weighted reference, B50, estimated B1500, and estimated ADC maps. The red contour indicates the prostate mask derived from the T2-weighted image. Supplementary Fig. S4 Qualitative comparison of diffusion-weighted images generated by DeDistortNet and comparative generative models in an extreme distortion case. Displayed images include the T2-weighted reference, B50, estimated B1500, and estimated ADC maps. The red contour indicates the prostate mask derived from the T2-weighted image. Supplementary Fig. S5 Reader study results across distortion severity. Before vs. after comparisons are shown for (a) geometric distortion, (b) anatomic delineation, and (c) need for reacquisition across mild, moderate, severe, and extreme distortion severity. In (a) and (b), lower scores indicate better image quality. In (c), “No*” denotes baseline-level artifact without diagnostic impact (Supplementary Table S2). Circle size is proportional to the number of cases with the same before–after rating combination. Supple [file 41747_2026_735_MOESM1_ESM.pdf]

Dual-conditioned diffusion model with anatomical guidance for geometric distortion correction in prostate MRI

ELECTRONIC SUPPLEMENTARY MATERIAL

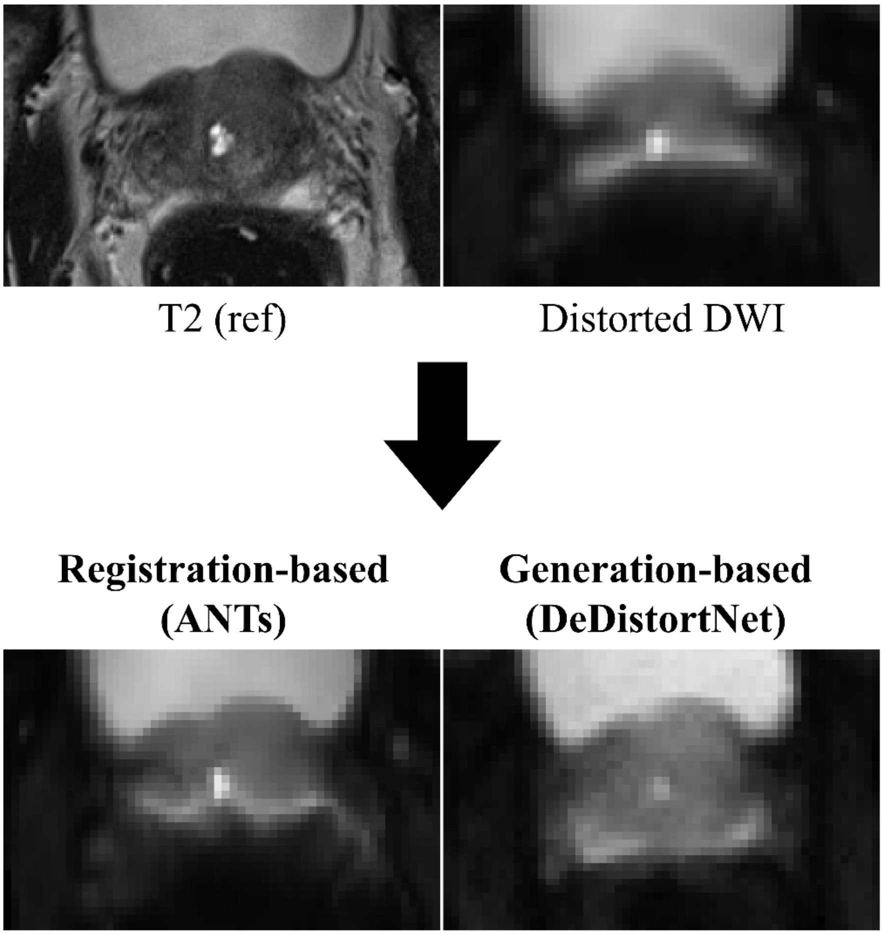

Supplementary Fig. S1 Comparison of distortion correction methods. The registration-based approach (e.g., advanced normalization tools) failed to correct severe distortion, whereas the proposed generative approach (DeDistortNet) successfully mitigated these artifacts.

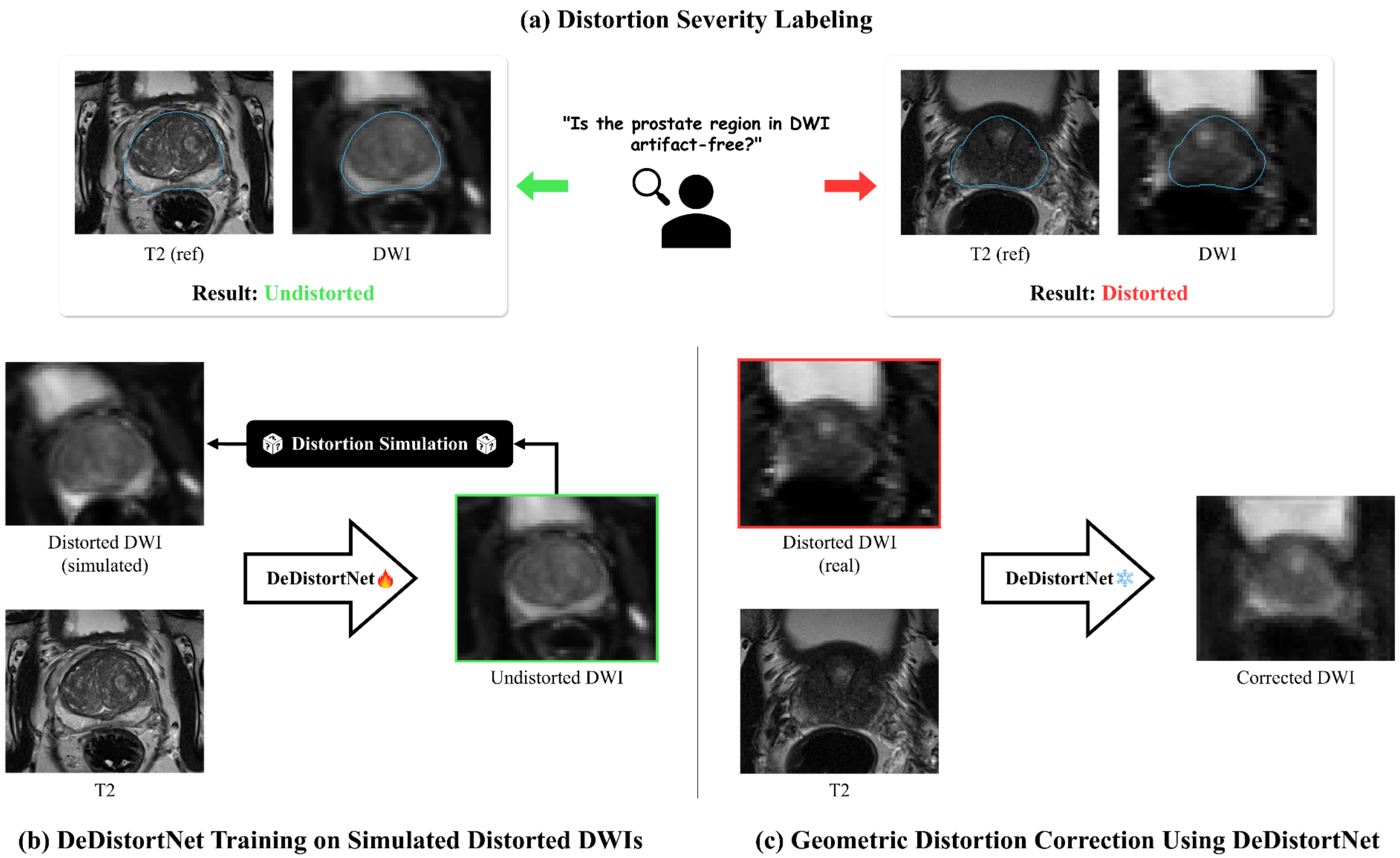

Supplementary Fig. S2 Overview of DeDistortNet workflow. (a) Severity labeling for diffusion-weighted images distortion. (b) Training procedure using simulated distorted diffusion-weighted images. (c) Geometric distortion correction using trained DeDistortNet.

Supplementary Table S1 Criteria for reader study.

Criterion	Description
**Geometric Distortion**	Degree of susceptibility-induced geometric distortion affecting the prostate gland.<br>**1:** No perceptible artifact<br>**2:** Minimal artifact without diagnostic impact<br>**3:** Mild artifact obscuring less than 50 percent of the peripheral zone<br>**4:** Moderate artifact obscuring more than 50 percent of the peripheral zone<br>**5:** Severe artifact affecting both peripheral and transitional zones
**Anatomic Delineation**	Clarity of the prostate gland and its boundaries relative to adjacent tissues.<br>**1:** Excellent delineation with strong contrast<br>**2:** Clear delineation with relatively clear contrast<br>**3:** Intermediate delineation with moderate contrast<br>**4:** Difficult delineation with subtle contrast<br>**5:** Poor delineation with insufficient contrast
**Need for Reacquisition**	Whether DWI reacquisition would be recommended in routine clinical practice.<br>**No**<br>**No*** (The artifact is at the baseline level and does not affect the diagnosis, so the answer is no.)<br>**Yes**

Supplementary Table S2 Dice similarity coefficients comparing prostate masks obtained from T2-weighted images and distorted/corrected diffusion-weighted images across varying distortion severities.

	No Distortion (n = 243)	Mild Distortion (n = 1,164)	Moderate Distortion (n = 816)	Severe Distortion (n = 298)	Extreme Distortion (n = 102)	Average (n = 2,623)
**Baseline**	***0.92 ± 0.07***	0.90 ± 0.08 *	0.87 ± 0.07 *	0.83 ± 0.09 *	0.73 ± 0.12 *	0.88 ± 0.09 *
**AdaIN**	0.33 ± 0.24 *	0.33 ± 0.24 *	0.35 ± 0.25 *	0.34 ± 0.24 *	0.32 ± 0.24 *	0.34 ± 0.24 *
**AdaConv**	0.36 ± 0.23 *	0.34 ± 0.24 *	0.37 ± 0.24 *	0.37 ± 0.24 *	0.32 ± 0.23 *	0.35 ± 0.24 *
**pix2pix**	0.89 ± 0.09 *	0.90 ± 0.09 *	0.91 ± 0.08 *	0.89 ± 0.09 *	0.91 ± 0.06 (p = 0.105)	0.90 ± 0.08 *
**CycleGAN**	0.82 ± 0.13 *	0.82 ± 0.13 *	0.83 ± 0.13 *	0.82 ± 0.13 *	0.83 ± 0.13 *	0.82 ± 0.13 *
**ControlNet**	0.90 ± 0.09 (p = 0.001)	0.91 ± 0.10 (p = 0.015)	0.91 ± 0.08 (p = 0.013)	0.90 ± 0.10 (p = 0.005)	0.91 ± 0.07 (p = 0.020)	0.91 ± 0.09 (p = 0.003)
**StableDiffusion**	0.88 ± 0.10 *	0.87 ± 0.11 *	0.88 ± 0.11 *	0.88 ± 0.12 *	0.87 ± 0.10 *	0.88 ± 0.11 *
**DeDistortNet**	0.92 ± 0.08 (p = 0.100)	0.92 ± 0.08 (p = 0.031)	0.93 ± 0.07 (p = 0.022)	0.92 ± 0.09 (p = 0.017)	0.93 ± 0.06 (p = 0.154)	0.92 ± 0.08 (p = 0.015)

Asterisks in each severity column indicate statistically significant differences ($p < 0.001$) versus the baseline no-distortion group based on Mann-Whitney *U* tests. Asterisks in the Average column indicate significant distributional differences across distortion severity groups based on Kruskal-Wallis tests.

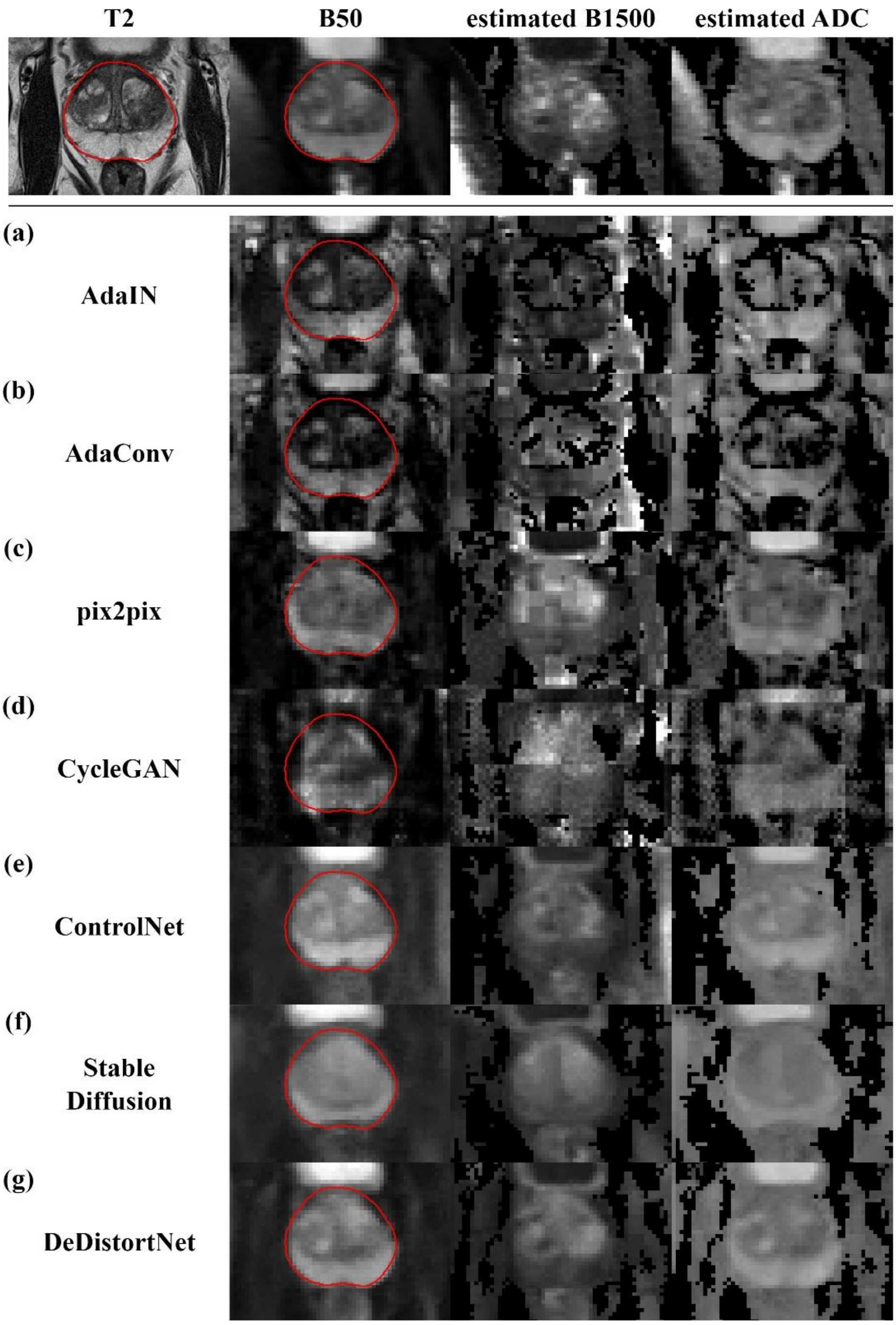

Supplementary Fig. S3 Qualitative comparison of diffusion-weighted images generated by DeDistortNet and comparative generative models in a no-distortion case. Displayed images include the T2-weighted reference, B50, estimated B1500, and estimated ADC maps. The red contour indicates the prostate mask derived from the T2-weighted image.

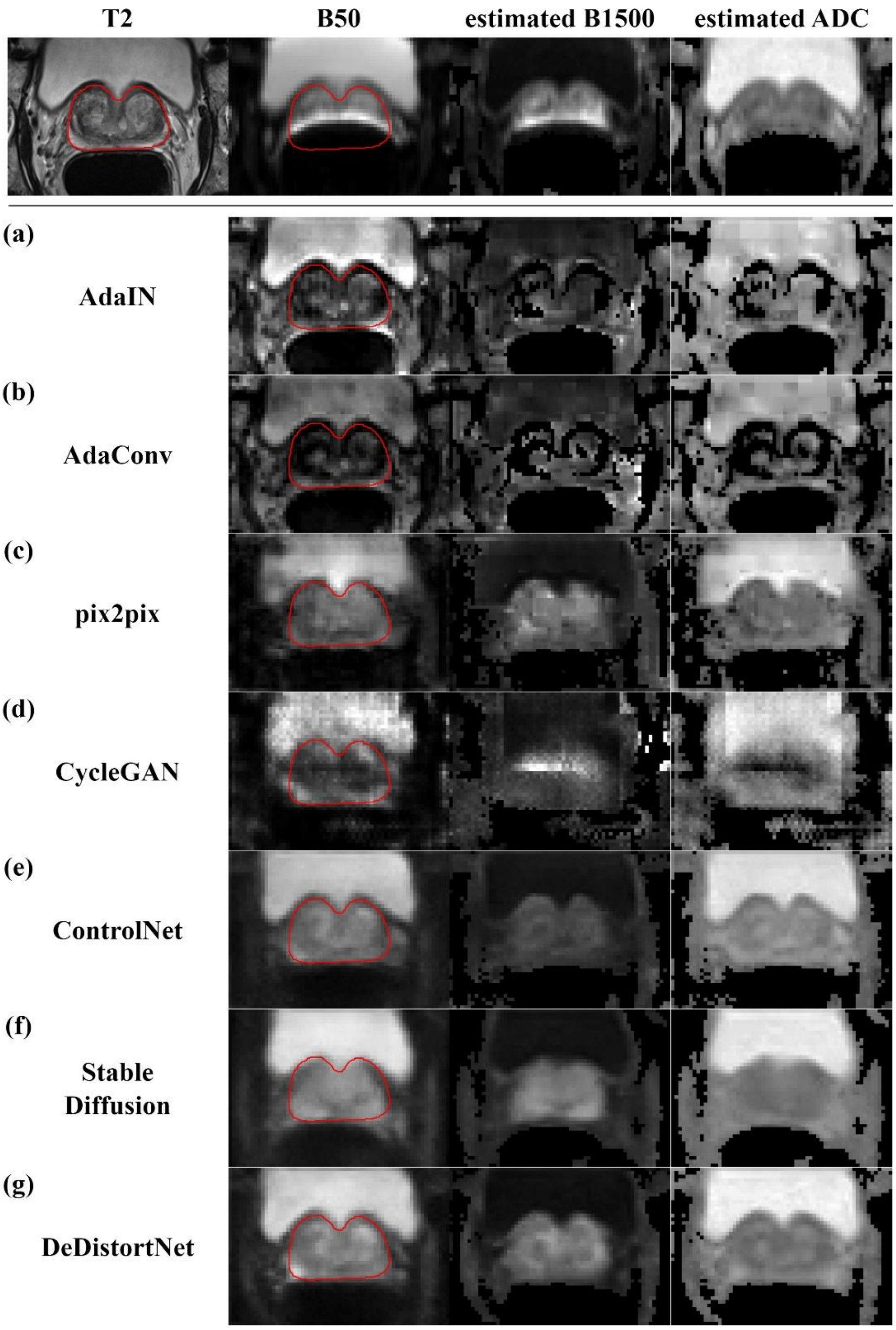

Supplementary Fig. S4 Qualitative comparison of diffusion-weighted images generated by DeDistortNet and comparative generative models in an extreme distortion case. Displayed images include the T2-weighted reference, B50, estimated B1500, and estimated ADC maps. The red contour indicates the prostate mask derived from the T2-weighted image.

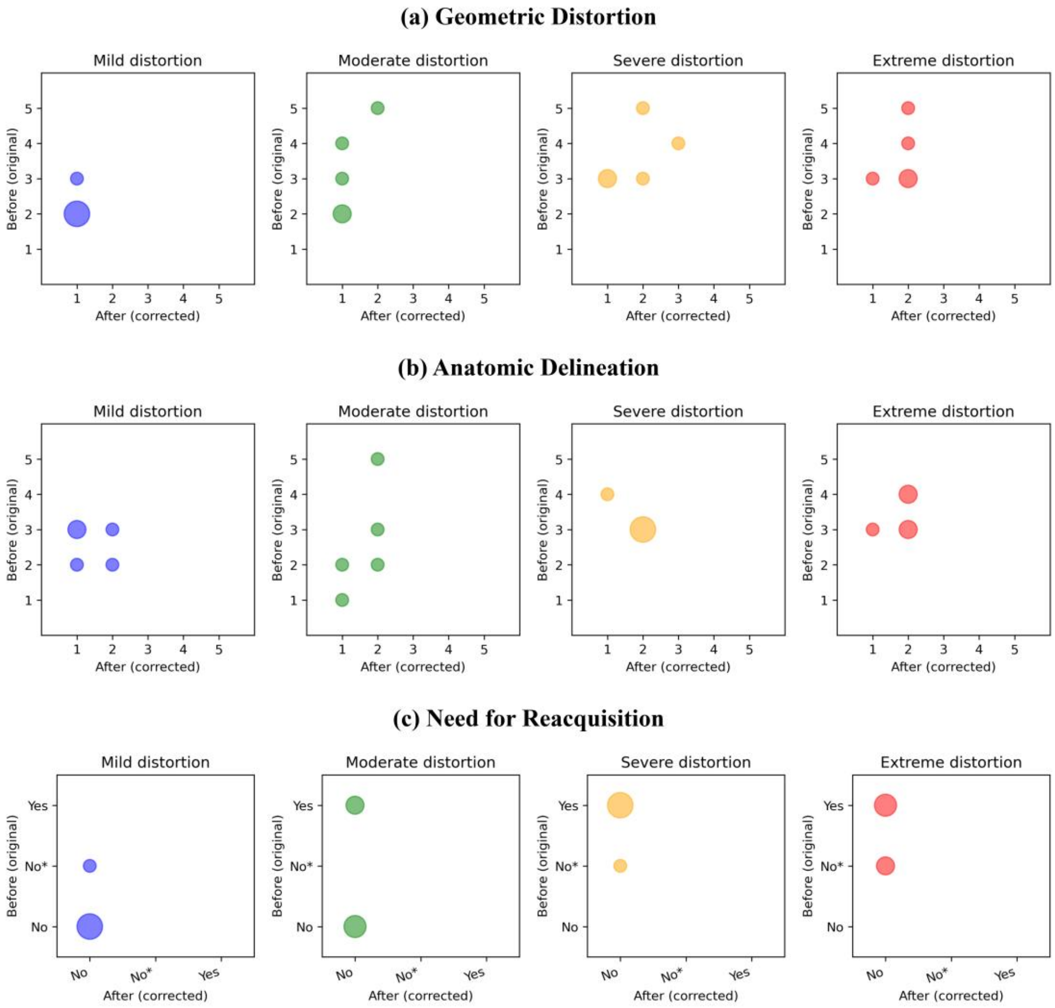

Supplementary Fig. S5 Reader study results across distortion severity. Before vs after comparisons are shown for (a) geometric distortion, (b) anatomic delineation, and (c) need for reacquisition across mild, moderate, severe, and extreme distortion severity. In (a) and (b), lower scores indicate better image quality. In (c), "No*" denotes baseline-level artifact without diagnostic impact (Supplementary Table S2). Circle size is proportional to the number of cases with the same before–after rating combination.
